# Supplementary material for: Dynamic structure of active nematic shells
Source: Nat Commun. 2016 Nov 21;7:13483. doi: 10.1038/ncomms13483 (PMC5121334; doi:10.1038/ncomms13483)
Supplement: Supplementary Information — Supplementary Figures 1-4 and Supplementary Notes 1-4 [file ncomms13483-s1.pdf]

Supplementary Figures

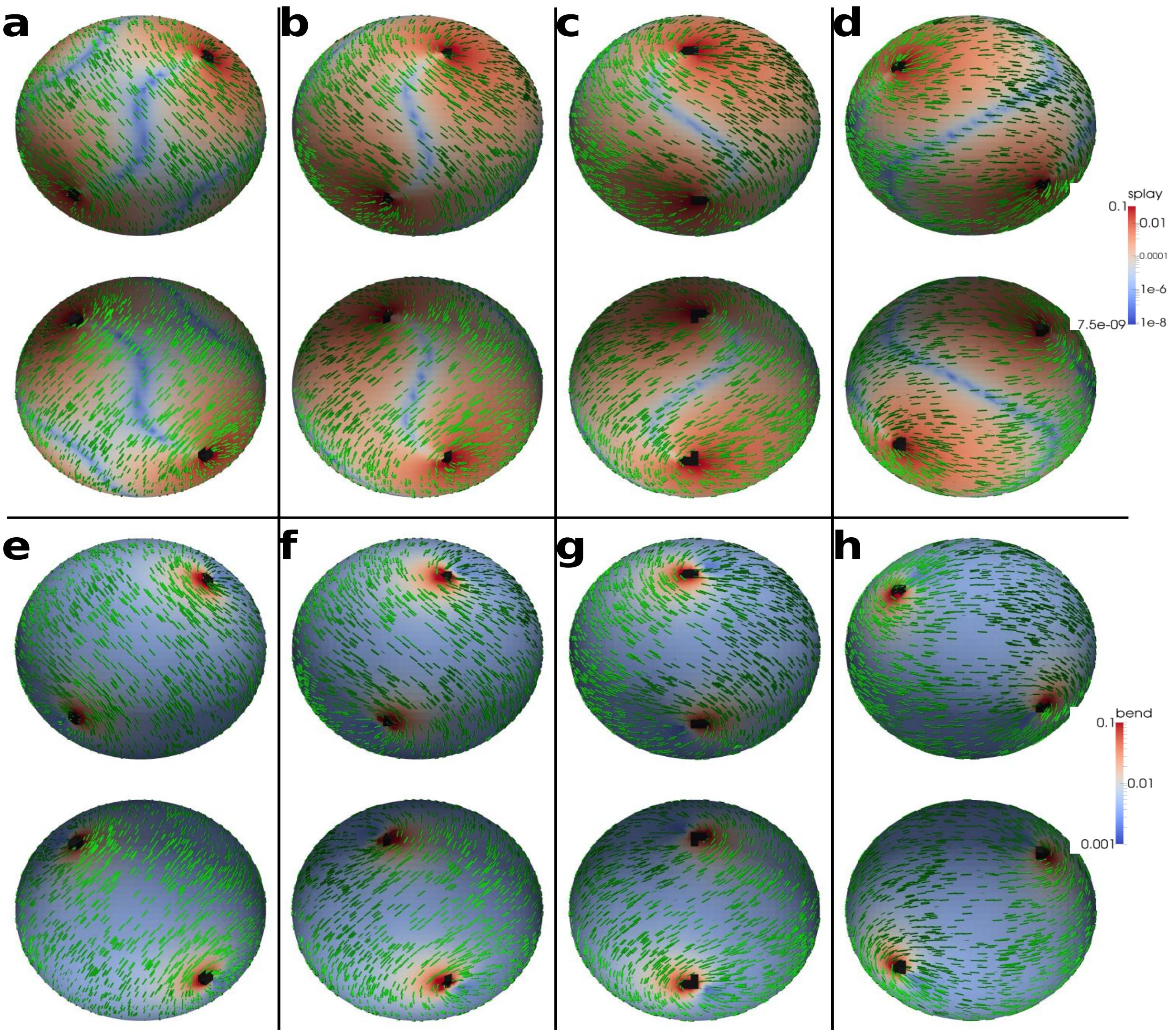

Supplementary Figure 1. **Elastic energy density distribution map**. Consecutive images of splay (**a**-**d**) and bend (**e**-**h**) energy density distribution during evolution for $\zeta = 0.001$. **a**, **e**, **d**, and **h** are in tetrahedral mode; **c** and **g** are in planar mode. Black regions denote defects. Green lines denote director field. Images in the same panel refer to the projections of opposite hemispheres. Energy density unit is $2.5\ J/m^3$.

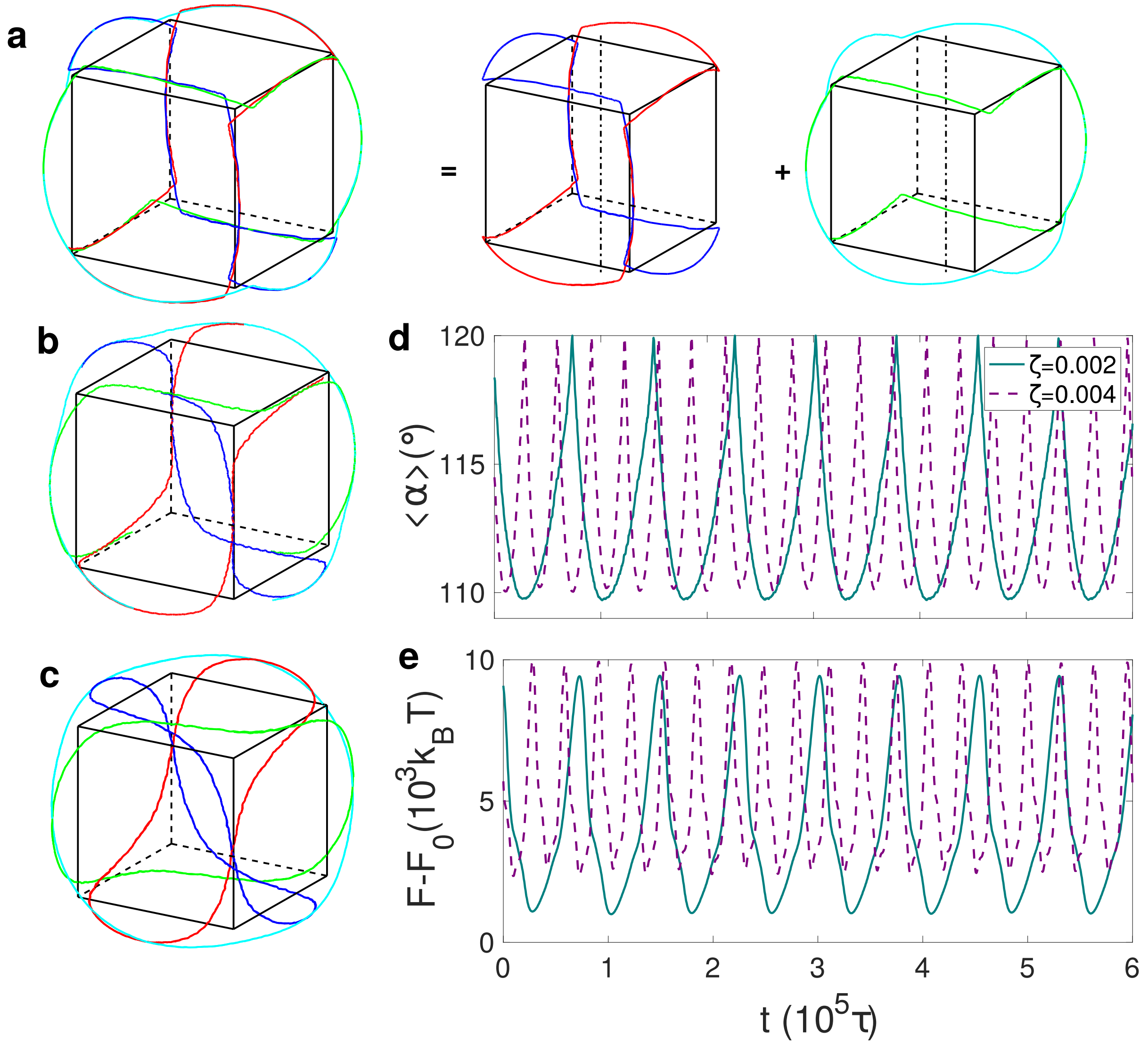

Supplementary Figure 2. **Defect trajectories and structures**. Closed defect trajectories for $\zeta = 0.0008$ (**a**), $\zeta = 0.002$ (**b**) and $\zeta = 0.004$ (**c**). A cubic is drawn as a guide to the eye. Individual zigzag defect trajectories trace a deformed cubic, as illustrated in (**a**). The two defects are symmetric about one symmetry axis of the cubic (dash-dotted lines). The average angular distance and free energy are plotted in (**d**) and (**e**), respectively.

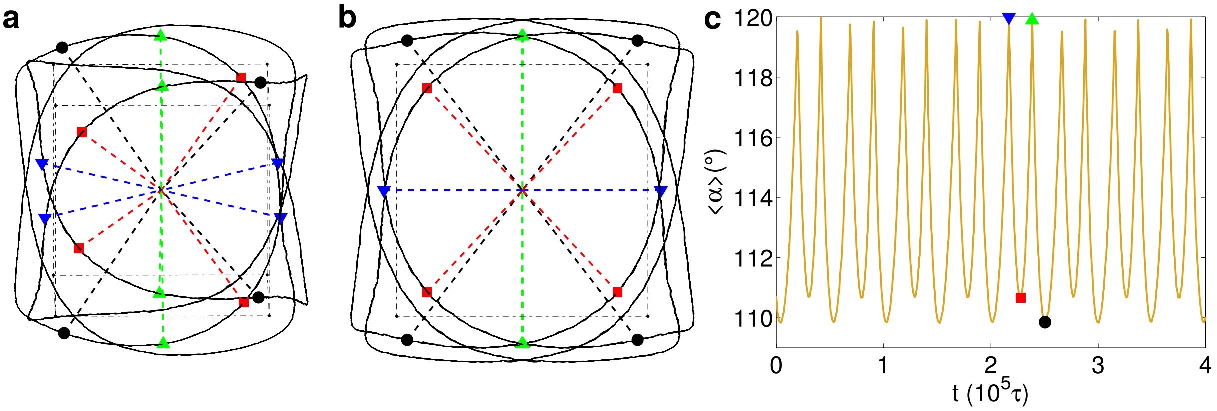

Supplementary Figure 3. **Evolution of defect configurations at intermediate activity**. Defect configurations at two view points for $\zeta = 0.005$ (**a**, **b**) and average angular distance plot (**c**). The four markers show defect structures at four different times indicated in **c**. **a** is a 3D view and **b** is viewing at -*y* direction. Defects are connected to origin by dashed lines. a cubic is shown in dash-dotted lines to guide eyes.

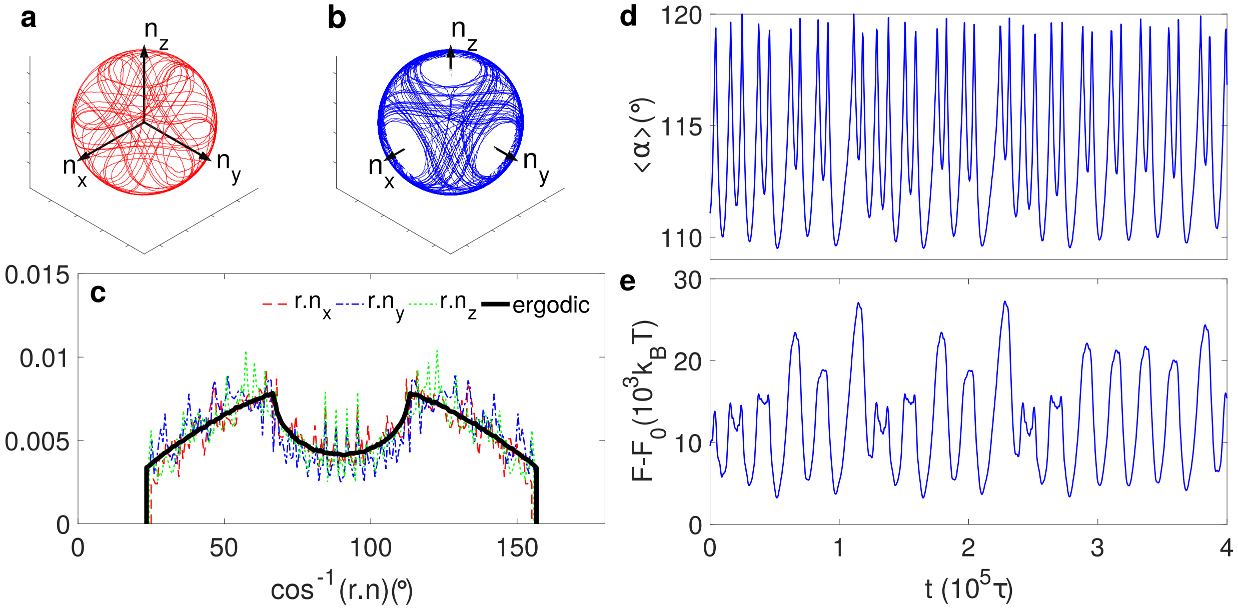

Supplementary Figure 4. **Evolution of defect structure at high activity**. Defect trajectory and structure are plotted for $\zeta = 0.01$. A single defect trajectory (**a**) and all defects' trajectories (**b**) on the shell. Defect position histograms (**c**) in terms of $cos^{-1}(\boldsymbol{r} \cdot \boldsymbol{n})$. n_x, n_y and n_z are the three symmetry axes of a cubic. Temporal behavior of average angular distance (**d**) and system's free energy (**e**}.

Supplementary Notes

Supplementary Note 1. Symmetry of Defect Configurations

In what follows, we show that if the four defects are symmetric about the symmetry axes of a deformed cubic, the following three equations hold: $\alpha_{12} = \alpha_{34}$, $\alpha_{13} = \alpha_{24}$ and $\alpha_{14} = \alpha_{23}$. Say defect 1 is located at $\mathbf{r_1} = (a, b, c)$ in Cartesian coordinates, and the three symmetry axes are $(1,0,0)$, $(0,1,0)$ and $(0,0,1)$. Thus the positions of the other 3 defects are $\mathbf{r_2} = (a, -b, -c)$, $\mathbf{r_3} = (-a, b, -c)$, and $\mathbf{r_4} = (-a, -b, c)$. Therefore

$$r_1 \cdot r_2 = r_3 \cdot r_4 = a^2 - b^2 - c^2,$$
$$r_1 \cdot r_3 = r_2 \cdot r_4 = -a^2 + b^2 - c^2,$$
$$r_1 \cdot r_4 = r_2 \cdot r_3 = -a^2 - b^2 + c^2.$$

Supplementary Note 2. Splay and Bend Distribution

In order to elucidate the origin of the periodic dynamics, in this section we analyze the spatial distributions of splay and bend energy densities. In Supplementary Fig. 1, we show the distributions of splay and bend energies at four selected times for $\zeta = 0.001$. These two energies are highly inhomogeneous in the vicinity of defects. High splay and high bend appear in opposite directions along the symmetry axis of the +1/2 defect. A band of low splay connects pairs of defects. That band deforms until the defects move away from each other, while the band forms within a different pair. A baseball-like band divides the surface. Such bands don't arise in the bend energy map. The intrinsic curvature of the shell induces a finite bend and hence it is more evenly distributed on the surface than splay. One can also appreciate in the figure that the size of the defects oscillates between two values. The defect is smallest when the defect velocity reaches a secondary peak, and when the splay energy reaches its first local maximum; the core size is the largest in the excited state, when the splay energy also reaches its maximum value.

Supplementary Note 3. Moderate Activity Dynamics

In Supplementary Fig. 2(a-c), several trajectories are shown for $\zeta = 0.0008, 0.002$ and 0.004. These trajectories also follow a deformed cube, but their shape depends on the activity; the trajectories for $\zeta = 0.0008$ are sharper than for higher activities. Different defect trajectories meet at the corners of the cube. When the defects of large- ζ systems move to the four corners of the deformed cube, for each pair the defect-defect distance is shorter than $\sqrt{8/3}R$, the edge length of an ideal tetrahedron circumscribed on a sphere of radius *R*. The corresponding value of $\langle\alpha\rangle_{min} = 109.7°$ for $\zeta = 0.002$ and $\langle\alpha\rangle_{min} = 110°$ for $\zeta = 0.004$ indicate that the state of minimum energy reached by the system deviates from the global ground state in which $\langle\alpha\rangle_{min} = 109.47°$ (see Supplementary Fig. 2(d)). In Supplementary Fig. 2(e) one can clearly see that min(F) is higher than that of a static system F_0. And, as shown in main text Fig. 5(a), there is a correlation between the minimum average angular distance and the minimum free energy that the system can attain. The higher $(F - F_0)_{min}$ is, the more pronounced the deviations become from a perfect tetrahedral state. As activity increases, the system of course moves further away from equilibrium, and the ``tetrahedral'' mode has a higher free energy. Main text

Fig. 5(b) describes the shape asymmetry (β) of the temporal evolution curves as a function of ζ. The temporal behavior of the angle $\langle\alpha\rangle$ and F are less ratchet-like for higher values of the activity. The energy difference between the two modes F_{planar} - F_{tetra} is smaller, and it is easier for the system to climb the energy barrier, leading to a less asymmetric temporal evolution.

Supplementary Note 4. High Activity Dynamics

If one further increases ζ, in the regime between $0.006 \leq \zeta \leq 0.01$, defects move in a less ordered manner, and their trajectories are no longer closed. Supplementary Fig. 4(a, b) shows these ``open'' trajectories for $\zeta = 0.01$. The defects still move in a pattern. As plotted in Supplementary Fig. 4(d, e), the system oscillates between $\langle\alpha\rangle \simeq 110°$ and $\langle\alpha\rangle \simeq 120°$ in a complicated way: a third mode appears in which $\langle\alpha\rangle$ reaches a minimum that is well above $109.47°$ when between two planar modes. The corresponding free energy also fluctuates in a random manner. If we measure the angles between the defect position **r** and the three symmetry axes of a cube (given by n_x, n_y and n_z), we see in Supplementary Fig. 4(c) that the defects are depleted within $23°$ off these axes. When the defect trajectories deviate from the deformed cube (as ζ increases), they remain near the sides of the cube, leading to a depletion near the axes regions. If one assumes that the defects are uniformly distributed outside of the depletion region, it is possible to generate a histogram that matches that predicted by our simulations to within statistical error (see Supplementary Fig. 4(c)), suggesting that defect positions for the disordered active system are ergodic. When $\zeta \geq 0.02$, momentum is no longer conserved and the four-defect structure is not stable.
